# Dihydroartemisinin inhibits EMT induced by platinum-based drugs via Akt–Snail pathway

**DOI:** 10.18632/oncotarget.21793

**Published:** 2017-10-10

**Authors:** Yuan Qin, Guang Yang, Meng Li, Hui-Juan Liu, Wei-Long Zhong, Xue-Qin Yan, Kai-Liang Qiao, Jia-Huan Yang, Deng-Hui Zhai, Wei Yang, Shuang Chen, Hong-Gang Zhou, Tao Sun, Cheng Yang

**Affiliations:** ^1^ State Key Laboratory of Medicinal Chemical Biology and College of Pharmacy, Nankai University, Tianjin, China; ^2^ Tianjin Key Laboratory of Molecular Drug Research, Tianjin International Joint Academy of Biomedicine, Tianjin, China

**Keywords:** dihydroartemisinin, EMT, Akt, Snail

## Abstract

Artemisinin and its derivatives exhibit a high activity against a range of cancer cell types both *in vitro* and *in vivo*. In clinical practice, platinum-based anti-cancer chemotherapy is widely used to treat tumors. However, a large proportion of patients receiving these treatments will relapse because of metastasis and drug resistance. The purpose of this study is to explore the combinational anti-metastatic effect of platinum-based drugs and dihydroartemisinin (DHA). Both DDP and oxaliplatin (OXA) at low doses could induce epithelial–mesenchymal transition (EMT) in HCC. Meanwhile, co-administration of DHA could enhance DDP and OXA chemosensitivity in HCC and reverse drug resistance. DHA reversed the morphological changes induced by DDP or OXA and reversed the changes in EMT biomarkers induced by DDP and OXA in HCC *in vitro* and *in vivo* via AKT–Snail signaling. DHA significantly increased platinum-based drug sensitivity and suppressed EMT induced by platinum-based drugs via AKT–Snail signaling in HCC. DHA is expected to become the new adjuvant for chemotherapy.

## INTRODUCTION

The era of platinum-based anti-cancer drugs was heralded by the clinical introduction of cisplatin, a square-planar platinum(II) complex whose anti-tumor properties were first reported by Rosenberg in 1969 [1]. Oxaliplatin (OXA), a third-generation platinum compound, exhibits promising activity against advanced HCC with tolerable toxicity [2]. A large population of HCC patients has been diagnosed with advanced disease; OXA-based chemotherapy is considered to be an important treatment choice for advanced-stage HCC [3]. However, biological deterioration after chemotherapy has recently been reported [4, 5]. Chemotherapeutic agents enhanced metastatic potential in HCC and other cancer cell types both *in vitro* and *in vivo*. In clinical practice, recurrence and metastasis after surgery, as well as poor chemosensitivity, persist as a major barrier to successful chemotherapy in HCC [6].

Accumulated evidence indicates that epithelial–mesenchymal transition (EMT) contributes to the “opposite effect” of platinum-based anti-cancer drugs [3, 7]. EMT is a process characterized by the loss of typical epithelial characteristics and the acquisition of mesenchymal traits [8]. Undergoing EMT progress, HCC cells lose the connection of cell–cell, the contact of cell–matrix, and normal epithelial polarity while gaining mesenchymal characteristics to migrate and invade the surrounding matrix. Recent studies suggested that EMT was closely related to drug resistance, and drug resistance cells could acquire EMT features.

Artemisinin and its derivatives are sesquiterpene lactones derived from the sweet wormwood (*Artemisia annua*), which has been used in Chinese traditional medicine for thousands of years as a remedy for fever and chills. Following their discovery and development as anti-malarial drugs by Tu Youyou’s group in the 1970s, artemisinin and its derivatives have come to represent the current front line in anti-malarial medicine [9]. Beyond these well-established antimalarial properties, there is accumulating evidence demonstrating that artemisinin and its derivatives possess cytotoxic effects against many human cancer cell types both *in vitro* and in animal experiments *in vivo* [10]. Depending on the cell line and experimental system, artemisinin affects a variety of processes in cancer cells, such as cell proliferation, apoptosis, and cellular hormone secretion [11]. Dihydroartemisinin (DHA), which is the most active derivative of artemisinin, has been recently reported to possess a preferential effect on cancer cells [12].

Prior studies have revealed that platinum-based anti-cancer drugs activated AKT signal pathway and induced EMT of cancer cells [13, 14]. Furthermore, latest data also show that DHA can suppress migration and invasion *in vitro* and *in vivo* in different cancer types via different pathways [15–18]. The aim of the present study was to examine the anti-tumor effect of DHA via inhibition of platinum-based drug-induced EMT and to explain the mechanism of DHA in the inhibition of the EMT.

## RESULTS

### DHA enhanced the effects of platinum-based drugs and reversed drug resistance in HCC

**Figure 1:**
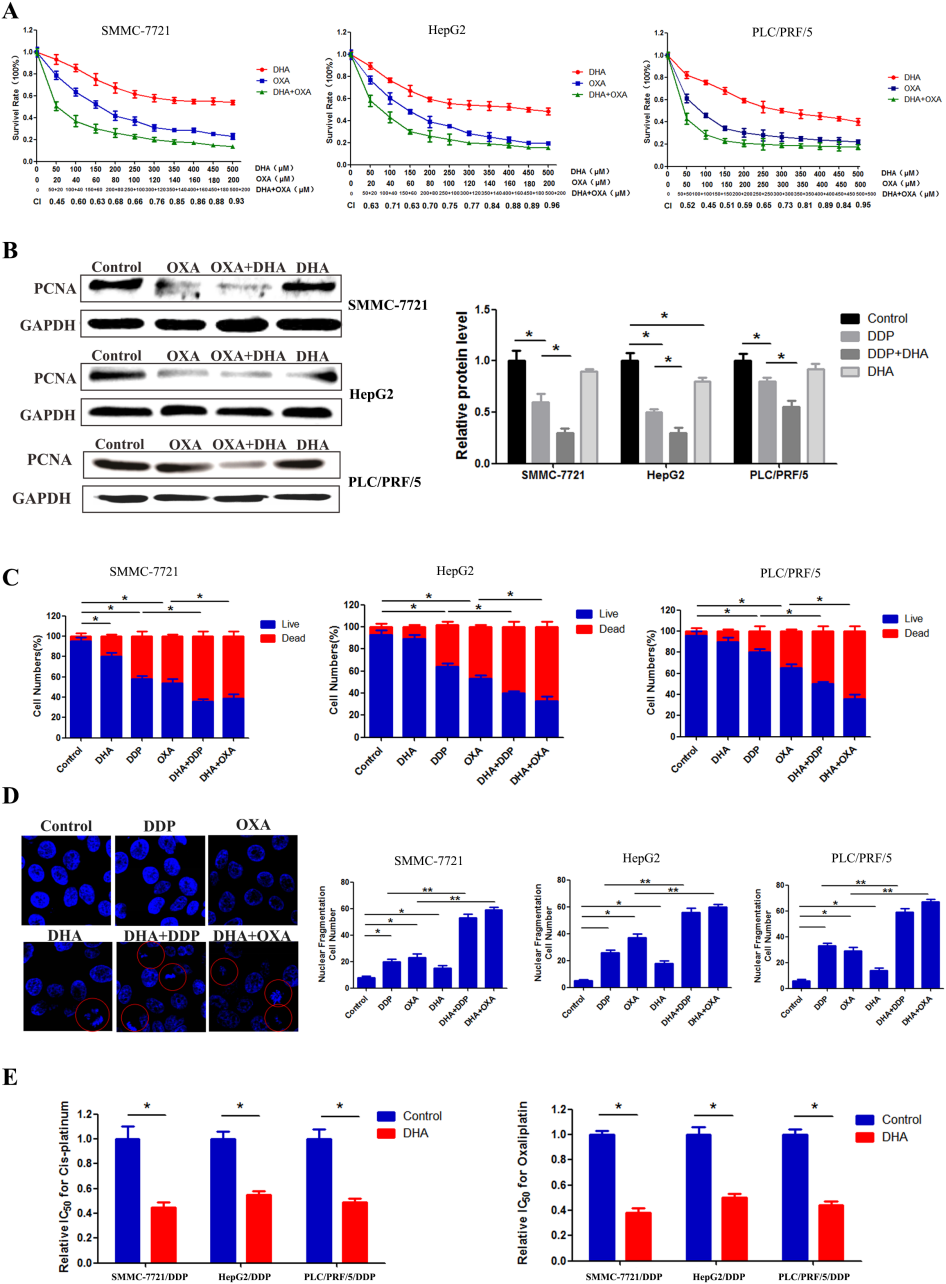
DHA enhanced the effects of platinum-based drugs and reverse drug resistance in HCC **(A)** Survival of SMMC-7721, HepG2 and PLC/PRF/5 cells treated with the indicated amounts of DHA or platinum-based drugs or DHA combined with platinum-based drugs. **(B)** The expression of the proliferation marker in different groups. **(C)** The proportion of dead cells in different groups. **(D)** The proportion of nuclear fragmentation in different groups. **(E)** The relative IC50 value of DDP-resistant SMMC-7721, HepG2 and PLC/PRF/5 for DDP and OXA with or without DHA. Data are presented as means of three experiments, and error bars represent standard deviation (*P < 0.05, ^**^P < 0.01).

Using an MTT assay, we determined the effect of a 48 h treatment with DHA, OXA, DDP, and DHA combined with OXA or DDP on cell viability. DHA, OXA, and DDP reduced cell viability in a dose-dependent manner. Meanwhile, DHA could evidently enhance the effects of DDP and OXA in inhibiting HCC cell proliferation. Moreover, Compusyn software analysis showed that the combination index (CI) value of combined treatment group was less than 1 at different doses, which indicated the synergistic effect between DHA and DDP or OXA as shown in Figure 1A. Western blot results showed that the combination treatment decreased the expression of proliferating cell nuclear antigen (PCNA), which is a marker of cell proliferation as shown in Figure 1B (DHA at 25 μM, DDP at 5 μM and OXA at 5 μM). Live/dead assay was used to explore the effect of DHA (25 μM ≈ 1/20 IC50), DDP (5μM ≈ 1/15 IC50) and OXA (5 μM ≈ 1/10 IC50) on HCC. The percentage of cell death in the combination treatment groups was significantly increased compared with the other groups, as shown in Figure 1C. Chromatin condensation and nuclear fragmentation were widely observed in the combination treatment groups compared with single application, indicating an apoptosis-inducing effect of the combination treatment group (DHA at 25μM, DDP at 5 μM, and OXA at 5 μM) as shown in Figure 1D. Moreover, DHA could reverse drug resistance of DDP and OXA in DDP-resistant cells(SMMC-7721/DDP, HepG2/DDP and PLC/PRF/5/DDP) as shown in Figure 1E.

### DHA inhibits migration and invasion induced by low dose DDP/OXA

**Figure 2:**
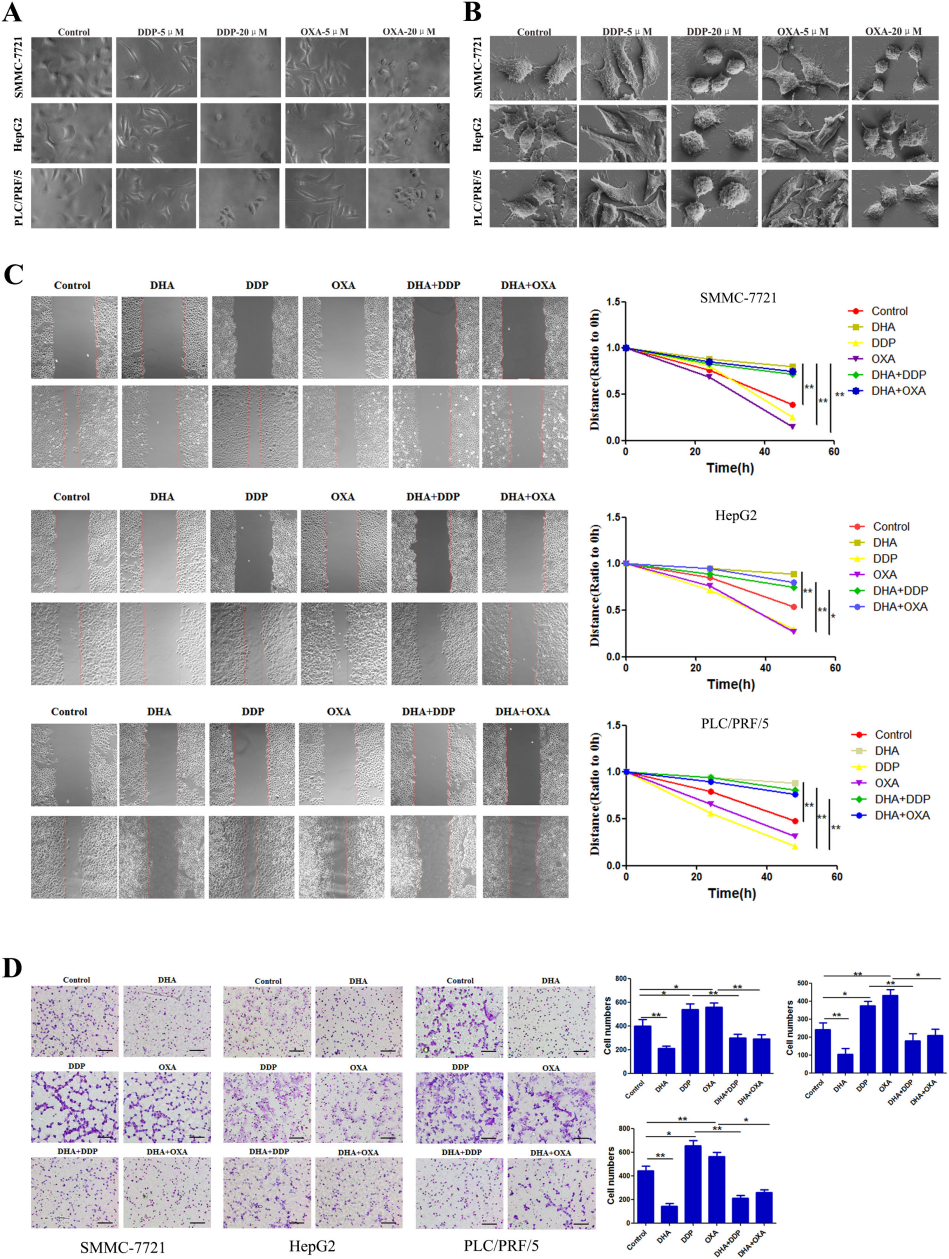
DHA inhibits migration and invasion induced by low dose DDP/OXA **(A)** Typical images of cells in different groups using an optical microscope. **(B)** Typical images of cells in different groups using a scanning electron microscope. **(C)** Cell viability was inhibited after re-incubation in different groups for 48 h. **(D)** Transwell chambers were utilized for the invasion assay, and images were obtained at 200× magnification. Data are presented as the means of three experiments, and error bars represent standard deviation (^*^P < 0.05, ^**^P < 0.01).

We further characterized the morphological changes with an optical microscope and a scanning electron microscope. As shown in Figure 2A and 2B, low doses of DDP and OXA led to the extension of pseudopodia and changes in microfilament structure. High doses of DDP and OXA showed characteristic features consistent with apoptosis, including cell rounding and detachment. To further investigate the motility potential associated with the combination treatment, we did an *in vitro* wound healing assay. After the 24 h and 48 h treatments, the migration ability of cells treated only with DDP and OXA had increased. Meanwhile, the wound gap was wider in the combination treatment groups, indicating that the combination treatment inhibits the motility of SMMC-7721, HepG2 and PLC/PRF/5 cells (Figure 2C). Using a Boyden chamber, we determined changes in cell invasiveness after 24 h. As shown in Figure 2D, DHA slightly reduced the number of cell invasions through the Matrigel-coated filter as compared to the control group. OXA and DDP increased the cell migration ability. The number of cell invasions through the Matrigel-coated filter was least in the combination treatment groups; therefore the combination treatment markedly inhibited the invasion of SMMC-7721, HepG2 and PLC/PRF/5 cells.

### DHA reverses changes in EMT biomarkers and inhibits AKT/Snail pathway induced by low dose DDP/OXA

**Figure 3:**
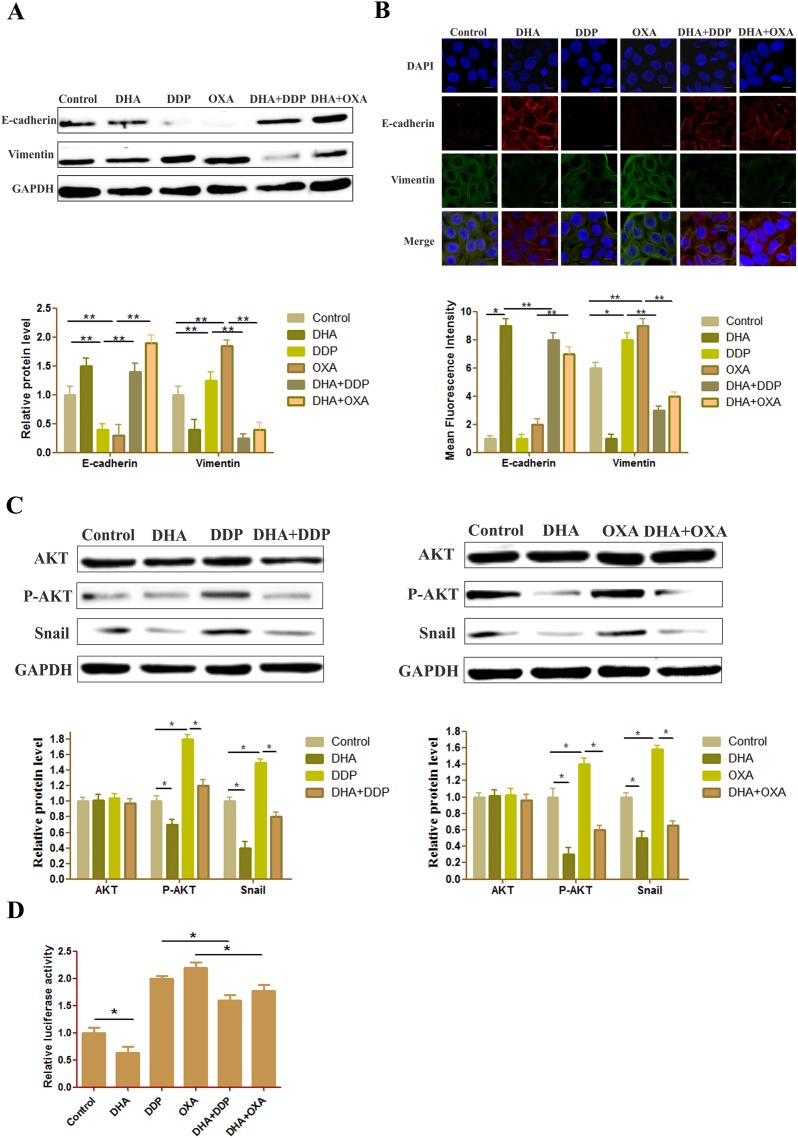
DHA reverses changes in EMT biomarkers and inhibits AKT/Snail pathway **(A)** Protein expression level of E-cadherin and vimentin in HepG2 cells treated with different drugs. The GAPDH blot served as the loading control. **(B)** Typical images of immunofluorescent double staining for E-cadherin and vimentin in HepG2 cells treated with different drugs. Each experiment was performed in triplicate. The results are the means of the three experiments, and error bars represent the standard deviation. **(C)** Protein expression levels of AKT and P-AKT in HepG2 cells treated with different drugs. The GAPDH blot served as a loading control. **(D)** Dual-luciferase assay results of snail gene expression in HepG2 cells treated with different drugs. Data are presented as the means of three experiments, and error bars represent standard deviation (^*^P < 0.05 and ^**^P < 0.01).

We examined the effect of the combination treatment on the expression of EMT biomarkers such as E-cadherin and vimentin. Western blot and immunofluorescence double staining results showed that the E-cadherin level has slightly decreased and the vimentin level has slightly increased in the DHA group. In the DDP and OXA groups, the EMT process has been promoted, the E-cadherin level decreased and vimentin level increased. However, in the combination treatment groups, the E-cadherin level significantly increased, and the vimentin level significantly decreased, demonstrating that the combination treatment effectively inhibited EMT (Figure 3A and 3B).

ATK is a core target that OXA can activate to induce EMT. Moreover, both artemisinin and its derivatives, e.g., DHA and artesunate, can inhibit the AKT pathway. Moreover, DHA can also prevent liver fibrosis by modulating the AKT pathway. The phosphorylation ATK, which was induced by DDP or OXA, was downregulated by DHA. Moreover, the expression of Snail was decreased in the combination treatment groups compared with the DDP or OXA treatment groups (Figure 3C). The results of the reporter gene assay also showed that DHA inhibited the level of Snail, whether it was used as a single application or in combination with platinum-based drugs (Figure 3D).

### DHA enhanced the anti-tumor effects of DDP and OXA in a mouse xenograft model

**Figure 4:**
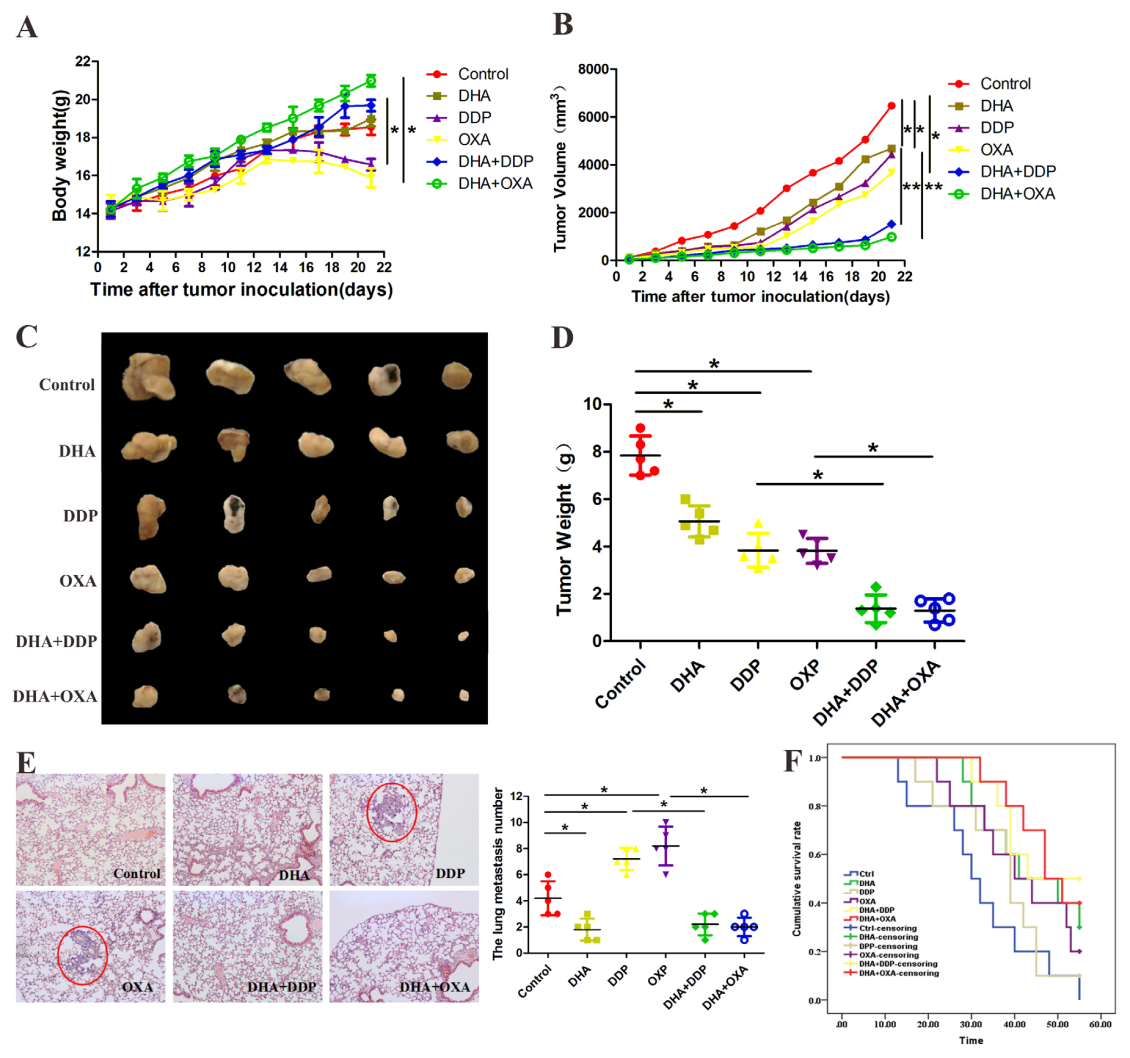
DHA enhanced the anti-tumor effects of DDP and OXA in a mouse xenograft model **(A)** Body weights (g) of animals with HepG2 xenografts. **(B)** Changes in the tumor volume of HepG2 xenografts. **(C)** Typical images of HepG2 xenografts. **(D)** Tumor weights (g) of animals with HepG2 xenografts. **(E)** The numbers of tumors that shifted to the lungs in different groups. **(F)** The median survival time of animals with HepG2 xenografts in different groups. Data are presented as the means of three experiments, and error bars represent standard deviation (^*^P < 0.05 and ^**^P < 0.01).

We next examined the effects of the combination treatment on HepG2 xenografts in BALB/c mice. Compared with the control group, DDP and OXA treatment groups’ body weight has reduced, and the combination treatment groups’ body weight has increased. Tumor growth was slightly suppressed in DDP and OXA groups, and significantly suppressed in the combination treatment groups. These results strongly suggest that the combination treatment inhibits tumor growth (Figure 4A and 4B). Compared with the other groups, the numbers of tumors that shifted stoves decreased after the combination treatment (Figure 4C). Survival curves showed that the combination treatment increased mice survival rate compared with the single application groups (Figure 4D)

### DHA alters EMT marker levels and inhibits the NF-κB/Snail pathway induced by low dose DDP/OXA in cancer tissues

**Figure 5:**
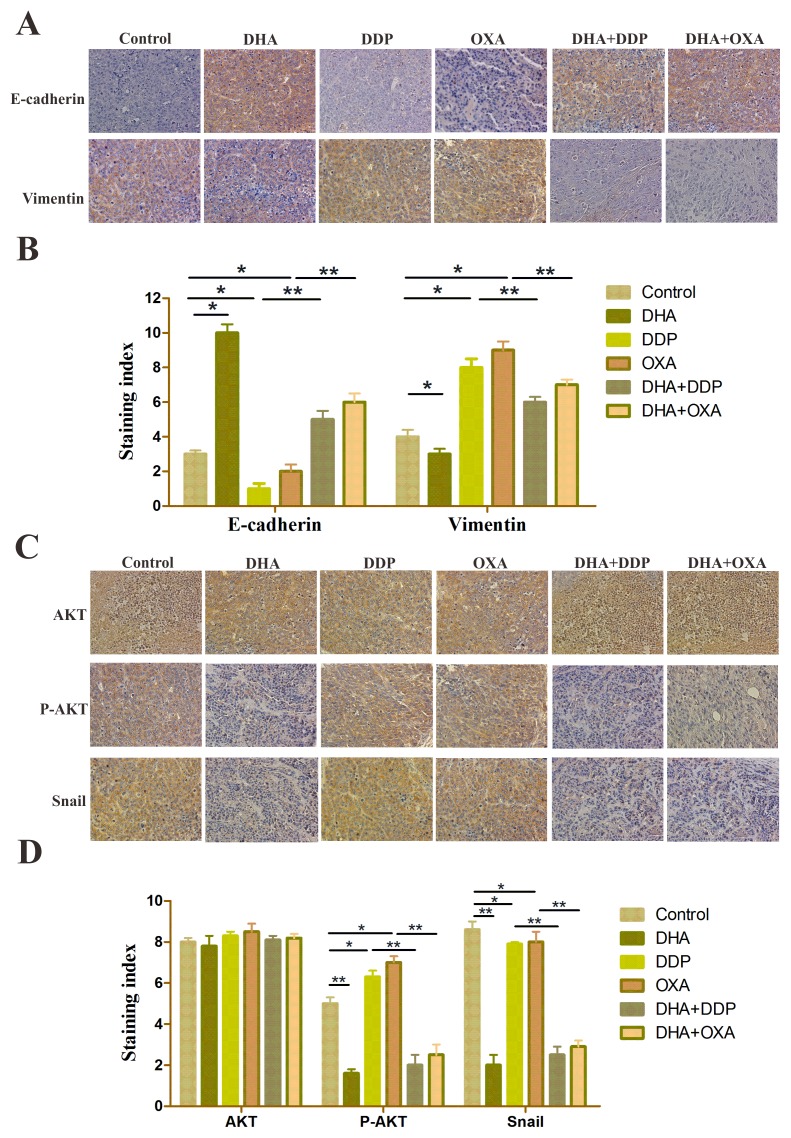
DHA alters EMT marker levels and inhibits the NF-κB/Snail pathway induced by DDP/OXA in cancer tissues **(A** and **C)** Representative immunohistochemical staining of tumor samples showing E-cadherin and vimentin-positive staining. **(B** and **D)** Representative immunohistochemical staining of tumor samples showed AKT, P-AKT, and snail-positive staining. Data are presented as the means of three experiments, and error bars represent standard deviation (^*^P < 0.05, ^**^P < 0.01).

**Figure 6:**
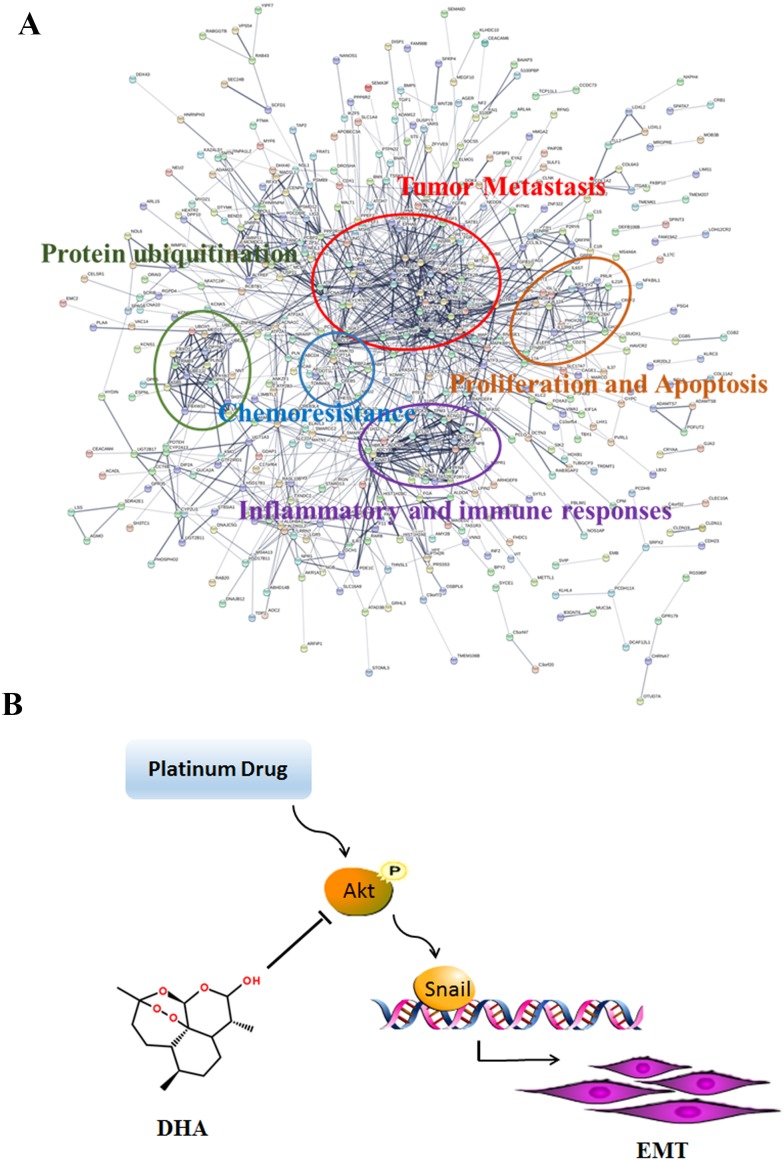
Effects of DHA in cancer cells **(A)** DHA affects biological processes in cancer cells. **(B)** A model showing the role of DHA in EMT induced by platinum drugs.

Expression of E-cadherin was enhanced in DHA and combination treatment groups relative to the control groups, and the combination group is more obvious. However, the vimentin level was increased in DDP and OXA treatment groups and decreased in the combination treatment groups (Figure 5A and 5C). Immunohistochemical staining for AKT, p-AKT and snail showed that the combination and the individual treatment groups had no significant difference in the total AKT levels. DDP and OXA treatments increased p-AKT and Snail levels, whereas DHA and combination treatments decreased p-AKT and Snail levels (Figure 5B and 5D). STRING database was selected to examine several types of interactions between the control and artemisinin groups. Many functions are influenced by DHA, including tumor metastasis, proliferation and apoptosis, and chemoresistance (Figure 6A). In conclusion, DHA significantly increased platinum-based drug sensitivity and suppressed EMT induced by platinum-based drugs via AKT–Snail signaling in HCC (Figure 6B).

## DISCUSSION

Although platinum-based anti-cancer chemotherapy is an important treatment choice for cancer, a large proportion of patients receiving these treatments will relapse [19, 20]. Many studies have been conducted to increase chemosensitivity in HCC patients. However, owing to little and limited understanding of chemoresistance mechanisms, the prognosis of HCC remains poor [21, 22].

Residual HCC after the OXA treatment demonstrated significantly increased metastasis *in vitro* and *in vivo* with the change of EMT markers and transcription factors [23, 24]. EMT induced by platinum-based anti-cancer drug is an important event contributing to the development of resistance to chemotherapeutics and induction of metastasis [25]. EMT is associated with proliferation, invasion/migration, and metastasis in HCC [26]. Moreover, EMT renders tumor cells more resistant to chemotherapeutic drugs via ABC proteins, which is considered a major target of EMT and a potential contributor to drug resistance [27, 28]. In accordance with previous reports, both DDP and OXA in low doses could alter cancer cells to EMT morphology and induce changes in EMT biomarkers in HCC *in vitro* and *in vivo*. Moreover, DDP and OXA increased the numbers of tumors that shifted stoves *in vivo* compared to the control group.

Since the first report of the possible anti-cancer properties of artemisinin in 1993, a large volume of studies have highlighted the potential of artemisinin and its derivatives as a novel therapeutic agent for cancer [29–31]. Artemisinin co-treatment synergizes with or enhances the effects of various conventional chemotherapy drugs in a range of cancer types. Co-administration of DHA could enhance DDP and OXA chemosensitivity in HCC. Moreover, DHA could reverse drug resistance of DDP and OXA in DDP-resistant HHC. Moreover, co-administration of DHA could inhibit EMT induced by DDP and OXA. DHA reverses the morphological changes induced by DDP or OXA. DHA reversed changes in EMT biomarkers induced by DDP and OXA in HCC *in vitro* and *in vivo*. The co-administration groups inhibited the numbers of tumors that shifted stoves *in vivo* compared to the simple chemotherapeutic medicine groups. Effective and safe artemisinin combinations could potentially have anti-cancer uses [31, 32].

AKT is a core target that OXA can activate to induce EMT. The OXA treatment could lead to a high level of reactive oxygen species (ROS). ROS induced by therapeutic agent contributes to the formation of the intramolecular disulfide bond of Akt and the increase of the phosphorylation level of Akt, which results in the activation of Snail, mTOR, and ERK, and the induction of EMT and drug resistance of cancer cells [23, 33–35]. Both DDP and OXA could increase the level of phosphorylation of AKT and activate the AKT–Snail pathway *in vitro* and *in vivo*. In addition, co-administration groups decreased the level of phosphorylation of AKT and inhibit the AKT–Snail pathway *in vitro* and *in vivo*. Gene expression microarray results showed that DHA could affect many biological processes including tumor metastasis, proliferation, apoptosis, and chemoresistance in cancer cells, which is in agreement with our results and other people’s findings [36, 37].

In conclusion, DHA significantly increased platinum-based drug sensitivity and suppressed EMT induced by platinum-based drugs via AKT–Snail signaling in HCC. DHA may be a potent platinum-based drugs sensitizer in HCC to improve the patients’ chemotherapy response.

## MATERIALS AND METHODS

### Cell culture

The HepG2, PLC/PRF/5 and SMMC-7721 human liver cancer cell line was from KeyGen Biotech. All cells were passaged and maintained in RPMI 1640 containing 10% fetal bovine serum (Hyclone, USA) and maintained at 37°C with 5% CO_2_ in a humidified atmosphere.

### Cell viability assay

Cell viability was determined by the 3-(4-5-dimethylthiazol-2-yl)-2, 5-diphenyltetrazolium bromide dye reduction assay (MTT assay). The cells (5 × 10^3^ cells/well) were seeded into each well of a 96-well flat bottom plate. After overnight incubation, the cells were treated with various concentrations of drugs and the cells were cultured at 37 °C with 5% CO_2_ in a humidified atmosphere. The cells were divided into five groups, as follows: (1) the DHA-treated group (0–500 μM), (2) the OXA-treated group (0–200 μM), (3) the DDP-treated group (0–500 μM), (4) the DHA + OXA-treated group, and (5) the DHA + DDP-treated group. After the drug treatment for 48 h, 20 μL MTT was added to each well and the cells were incubated for 4 h. Following the subsequent removal of the supernatant, 150 μL dimethyl sulfoxide (DMSO) was added to dissolve the formazan crystals. Absorbance was measured at 590 nm with a microplate reader (Multiskan™ FC, Thermo Scientific, Waltham, MA, USA).

### Wound-healing assay

HepG2, PLC/PRF/5 and SMMC-7721 human liver cancer cells (5 × 10^5^ cells/well) were grown to confluency in 48-well plates. Cell monolayers were scratched using a 200 μL pipette tip to create a wound and then washed once with phosphate-buffered saline (PBS). The cells were divided into six groups, as follows: (1) the control group (solvent control), (2) the DHA-treated group (25 μM), (3) the OXA-treated group (5 μM), (4) the DDP-treated group (5 μM), (5) the DHA + DDP-treated group (25 μM DHA + 5 μM DDP), and (6) the DHA + OXA-treated group (25 μM DHA + 5 μM OXA). Photographs were taken at 24 and 48 h with a Nikon microscope.

### Invasion assays

The invasive potential of HepG2, PLC/PRF/5 and SMMC-7721 human liver cancer cells was determined by a Matrigel invasion assay using polycarbonate membranes (8.0 μm pore size) (Corning, USA) in the upper chamber of 24-well Transwell culture chambers coated with Matrigel. Cells (2 × 10^5^ cells/mL) suspended in 200 μL of a serum-free medium were placed in the upper chambers, and the lower chambers were filled with RPMI 1640 with 10% fetal bovine serum as the chemoattractant. The cells were divided into six groups as described in the wound-healing assay. After the incubation at 37°C for 24 h, cells on the upper surface of the filter were removed using a cotton swab. Cells migrating or invading through the filter to the lower surface were fixed with 4% paraformaldehyde for 20 min and stained with 0.1% crystal violet for 10 min. Migrated or invaded cells were viewed and photographed under a phase-contrast microscope and counted in five fields (at 100× magnification).

### Immunofluorescence assays

HepG2 human liver cancer cells (2 × 10^5^ cells/mL) were seeded into 24-well plates. The cells were divided into six groups as described in the wound-healing assay. After the incubation for 24 h, cells were washed twice in PBS, fixed in 10% cold formalin (−20°C), and then blocked with FBS (5% BSA and 0.1%Tritonx-100) for 1 h. The cells were then incubated in the same solution containing primary antibodies specific for E-cadherin (1:100 dilution) and vimentin (1:100 dilution) for 1 h at room temperature. After three washes with phosphate-buffered saline (PBS), the cells were incubated in a secondary antibody (1:200 dilution) for 30 min at room temperature, incubated with DAPI for 10 min at room temperature, washed twice with PBS, and then observed using a high-content screening (HCS) system (Thermo Fisher, USA).

### Western blot analysis

HepG2 human liver cancer cells were grown to confluence in 6-well plates and grown to 40–50% confluency. The cells were divided into six groups, as described in the wound-healing assay. After incubation for 24 h, the cells were washed twice with cold PBS and then harvested with a rubber scraper. After centrifugation, 50 μg of total cellular lysates was mixed with SDS sample buffer, boiled for 10 min and then subjected to electrophoresis on 12% SDS–polyacrylamide gels. Proteins in each gel were transferred to PVDF membranes (Millipore, Billerica, MA, USA). blocked with TBST containing 5% skim milk powder, and then incubated overnight at 4 °C with primary antibodies against E-cadherin (1:1000), vimentin (1:1000), and GAPDH (1:3000). The membranes were washed with TBST thrice for 10 min each and incubated with an appropriate HRP-conjugated secondary antibody for 1 h at room temperature. Protein bands were detected by the enhanced chemiluminescence detection system.

### Cell activation and intracellular staining

HepG2, PLC/PRF/5 and SMMC-7721 human liver cancer cells (20,000 cells/well) were seeded in each well of a 96-well plate. The cells were divided into six groups as described in the wound-healing assay. Cells were collected and washed with phosphate buffer saline. Staining with LIVE/DEAD Fixable Dead Cell Stain Kit (Molecular Probes) was performed before fixation to allow gating on viable cells. The percentage of live and dead cells was determined by flow cytometry.

### Scanning electron microscopy (SEM)

Cells were seeded into 24-well plates and grown to 40–50% confluency. After overnight incubation, the cells were divided into five groups: (1) the control group (solvent control), (2) the DDP-treated group (5 μM), (3) the DDP-treated group (20 μM), (4) the OXA-treated group (5 μM), and (5) the OXA-treated group (20 μM). After incubating for 24 h, the cells were fixed and dehydrated in acetone/isoamyl acetate (1:1) and dried with a gradient concentration of acetonitrile. The samples were pasted onto the SEM Sample Stub using a carbon tape and the sample was coated with gold for 40 s and analyzed under a high-resolution scanning electron microscope (LEO 1530 VP, Germany).

### Animal studies

A total of 30 male BALB/c mice (18–20 g) were purchased from the National Institute for Food and Drug Control or experimental animal center of the Military Medical Science Academy of the Chinese PLA. All animals in this experiment were well cared for. HepG2 xenografts of tumors (1 × 10^6^/ml) suspended in PBS were established by a subcutaneous injection into the flank. One day after tumor cell inoculation, the mice were randomly divided into 6 groups (*n* = 5/group) with identical average body weight. After the tumors reached an approximate volume of 100 mm^3^, the mice were divided into six groups, as follows: (1) the control group (saline by oral gavage once a day), (2) the DHA group (40 mg/mL), (3) the OXA group (5 mg/mL), (4) the DDP group (5 mg/mL), (5) the DHA + DDP group (40 mg/mL DHA + 5 mg/mL DDP), and (6) the DHA + OXA group (40 mg/mL + 5 mg/mL). The volumes of the transplanted tumor were measured every day. At the end of the experiments, the body weights of mice were recorded and the tumor and lung tissues of mice were collected and stained with (H&E).

### Immunohistochemical analysis

Immunohistochemical detection was performed on paraffin-embedded slides of tumor sections. These procedures employed a previous method described by Qin et al [12]. The Akt, p-Akt, E-Cadherin, and vimentin antibodies were diluted to 1:100; PBS replaced the first antibody as the negative control. The positive antibody reaction was scored into four grades, according to the staining intensity, as follows: none (0), weak brown (1+), moderate brown (2+), and strong brown (3+). The percentages of positive cells were divided into five classes based on the percentage of tumor cells stained: 0 for no cells, 1 for 1%–25%, 2 for 25%–50%, 3 for 50%–75%, and 4 for >75%.

### Multidimensional liquid chromatography-tandem mass spectrometry

HepG2 cells (4 × 10^3^ cells/mL) were seeded in a 100 mm dish to 70–80% confluence. The cells were then cultured in the presence (25 μM) or absence of artemisinin for 24 h. After cell lysis, samples were tested by using multidimensional liquid chromatography-tandem mass spectrometry.

### Statistical analysis

All data are expressed as means ± standard deviation. Comparisons between groups were performed by one-way analysis of variance followed by Bonferroni post hoc test (SPSS software package version 17.0, SPSS Inc., Chicago, IL, USA). The level of significance was set at *P* < 0.05.
